# Clinical uses of Bupropion in patients with Parkinson’s disease and comorbid depressive or neuropsychiatric symptoms: a scoping review

**DOI:** 10.1186/s12883-022-02668-4

**Published:** 2022-05-05

**Authors:** Matteo Vismara, Beatrice Benatti, Gregorio Nicolini, Ilaria Cova, Edoardo Monfrini, Alessio Di Fonzo, Vincenza Fetoni, Caterina A. Viganò, Alberto Priori, Bernardo Dell’Osso

**Affiliations:** 1 Department of Mental Health, Department of Biomedical and Clinical Sciences Luigi Sacco, Luigi Sacco Hospital, University of Milan, Via G.B. Grassi, 74, 20157 Milan, Italy; 2 “Aldo Ravelli” Center for Neurotechnology and Brain Therapeutic, University of Milan, Milan, Italy; 3 Neurology Unit, Luigi Sacco University Hospital, Milan, Italy; 4 Dino Ferrari Center, Neuroscience Section, Department of Pathophysiology and Transplantation, University of Milan, Milan, Italy; 5 Foundation IRCCS Ca’ Granda Ospedale Maggiore Policlinico, Neurology Unit, Milan, Italy; 6 Neurology Department, ASST Fatebenefratelli Sacco, Milan, Italy; 7 Neurology Department of Health Sciences, San Paolo University Hospital, ASST Santi Paolo e Carlo, University of Milan Medical School, Milan, Italy; 8 Department of Psychiatry and Behavioral Sciences, Bipolar Disorders Clinic, Stanford University, Stanford, CA USA; 9 “Centro per lo studio dei meccanismi molecolari alla base delle patologie neuro-psico-geriatriche”, University of Milan, Milan, Italy

**Keywords:** Bupropion, Parkinson’s disease, Depression, Neuropsychiatric symptoms, Pharmacological treatment

## Abstract

**Objective:**

Bupropion, an antidepressant inhibiting the reuptake of dopamine and noradrenaline, should be useful to treat depressive symptoms in patients with Parkinson’s disease (PD). Limited and conflicting literature data questioned its effectiveness and safety in depressed PD patients and extended its use to other neuropsychiatric symptoms associated with this disorder.

**Design:**

The databases PubMed, Embase, Web of Sciences, Cochrane Library, and the grey literature were searched. Following a scoping review methodology, articles focusing on Bupropion uses in PD patients who manifested depressive or other neuropsychiatric alterations were reviewed.

**Results:**

Twenty-three articles were selected, including 7 original articles, 3 systematic reviews or meta-analyses, 11 case reports, 1 clinical guideline, and 1 expert opinion. Bupropion showed considerable effectiveness in reducing depressive symptoms, particularly in relation to apathy. Solitary findings showed a restorative effect on compulsive behaviour secondary to treatment with dopamine as well as on anxiety symptoms. The effect on motor symptoms remains controversial. The safety profile of this medication seems positive, but additional precautions should be used in subjects with psychotic symptoms.

**Conclusion:**

The available literature lacks good evidence to support the use of Bupropion in PD patients presenting depressive symptoms. Further investigations are needed to extend and confirm reported findings and to produce accurate clinical guidelines.

## Background

Motor symptoms are the cardinal manifestation of Parkinson’s disease (PD), however, the clinical picture typically also manifests with non-motor symptoms like neuropsychiatric alterations, autonomic dysfunctions, sleep disturbances, sensory deficits, and cognitive impairment [1, 2]. Non-motor symptoms often anticipate the diagnosis of PD and their underrecognition might lead to delay in the correct diagnosis and treatment [3]. Additionally, the frequent overlap between neurological and psychiatric symptoms complicates the course of the illness and remains a real challenge in terms of differential diagnosis, management, and treatment approach [4, 5].

In PD patients, depressive symptoms are common, with a prevalence that varies from 35 to 50% of cases [6, 7] and are associated with greater disability, rapid progression of motor symptoms, and increased mortality [8, 9]. Despite the high prevalence, depressive symptoms remain frequently undiagnosed because they often mimic those of PD [10]. Among different therapeutic strategies to target depressive symptoms in PD, antidepressants are the most used. However, the effectiveness and safety of these medications, particularly of “newer” compounds (beyond the selective serotonin reuptake inhibitors (SSRIs) and the serotonin–norepinephrine reuptake inhibitors (SNRIs)), is supported by limited scientific evidence [11, 12].

In the list of “newer” antidepressants, Bupropion has been described as a potential option for the treatment of depressive symptoms in PD [13]. Compared to other classes of antidepressants, Bupropion has a unique mechanism of action targeting the dopaminergic and noradrenergic systems (through inhibition of the reuptake of these two neurotransmitters) [14] whose alterations are at the core of PD pathogenesis [15]. Indeed, among other etiopathogenic factors responsible for depressive symptoms in PD, a specific loss of dopamine and noradrenaline innervation of cortical and subcortical components of the limbic system has been proposed. Hypofunction of these neurotransmitters might cause apathy, loss of interest, pleasure, and energy, impaired executive function, and concentration disturbances, that might be independent of a comorbid depressive disorder [15].

Remarkably, Bupropion does not affect the serotonergic system (with fewer side-effects such as drowsiness, weight gain, and sexual dysfunction) and potentially increases PD patients’ adherence to antidepressant treatment [13]. For the same reason, the combination of Bupropion with monoamine-oxidase inhibitors (frequently prescribed in PD) is likely associated with an abated risk of serotonin syndrome [11]. Lastly, a neuroprotective effect of Bupropion in patients with PD has been hypothesized, being possibly mediated by a reduction of dopaminergic toxicity in intracytoplasmic/extravesicular compartments [16].

Despite these theoretical properties, Bupropion efficacy on depressive symptoms in PD has been reported only in limited clinical investigations [17–19]. This presumably reflects the clinical practice that favors the use of other antidepressants (i.e., SSRIs or SNRIs) which are prescribed as first-line treatment in Major Depressive Disorder [20]. Moreover, considering the Bupropion effect on the dopaminergic system, some concerns might be raised about its safety and tolerability. In this respect, the available clinical data showed controversial results. On one hand, Bupropion has been shown to ameliorate PD-related motor symptoms [21], while, on the other, some studies underlined the risk of Bupropion-induced movement disorders [22–24]. Apart from depressive symptoms, some investigations showed a positive effect of Bupropion on other neuropsychiatric manifestations often characteristic of individuals suffering from PD, for example, drowsiness and sleep problems [25, 26].

Considering this background, we primarily sought to review the current knowledge on the effectiveness and safety of Bupropion in patients with PD and comorbid depressive symptoms. Secondarily, we aimed to extend the knowledge on Bupropion additional uses in PD patients who manifested other neuropsychiatric alterations. This work primarily aimed to map and characterize the current literature on this topic. Additionally, it will help to draft specific recommendations which could guide clinicians to manage neuropsychiatric manifestations in patients with PD and, eventually, to delineate a PD patient’s profile more appropriate for treatment with Bupropion.

## Methods

A scoping review method was deemed appropriate for our research question considering the lack of univocal findings and the paucity of data on Bupropion use in patients with PD [27]. We followed the modified scoping review procedures as outlined by Arskey and O’Malley [28] and further elaborated by Levac and colleagues [27]. Accordingly, the present review followed the proposed five-stage methodological framework: developing the research question, identifying relevant articles, selecting articles, extracting data, and collating results (the sixth step - engaging stakeholders through consultation - was not adopted, as considered optional by the above-mentioned approach [28]).

### Definitions and search strategies

The authors discussed and reached a consensus on the research question and the target of the investigation. The research question consisted of investigating the therapeutical uses of Bupropion in patients with a diagnosis of PD (or parkinsonism) who manifested depressive symptoms or other neuropsychiatric alterations (e.g., sleep disturbances, anxiety symptoms). Therefore, we included studies where Bupropion was primarily prescribed as an antidepressant (considering its main clinical indication) but also studies that investigated its use for potentially any other therapeutical reason in PD patients. Considering scoping review’s methodology, we decided to search for studies that differed on several variables, including sample characteristics, Bupropion dosage, or outcomes investigated.

To identify relevant studies, we searched electronic databases PubMed, Embase, Web of sciences, Cochrane Library from inception to May 2021. An inclusive search strategy was performed using the terms “Bupropion” AND “Parkinson” OR “parkinsonism”. Considering the original generic name of Bupropion (i.e., amfebutamone), an additional research was performed using this term. To identify further references not captured in the published medical literature, we also searched Opengrey (SIGLE) and Google Scholar, screening the first 100 results for relevance to our clinical question. Additional articles potentially relevant to our objectives were identified through reviewing reference lists of selected articles.

### Study selection

**Fig. 1:**
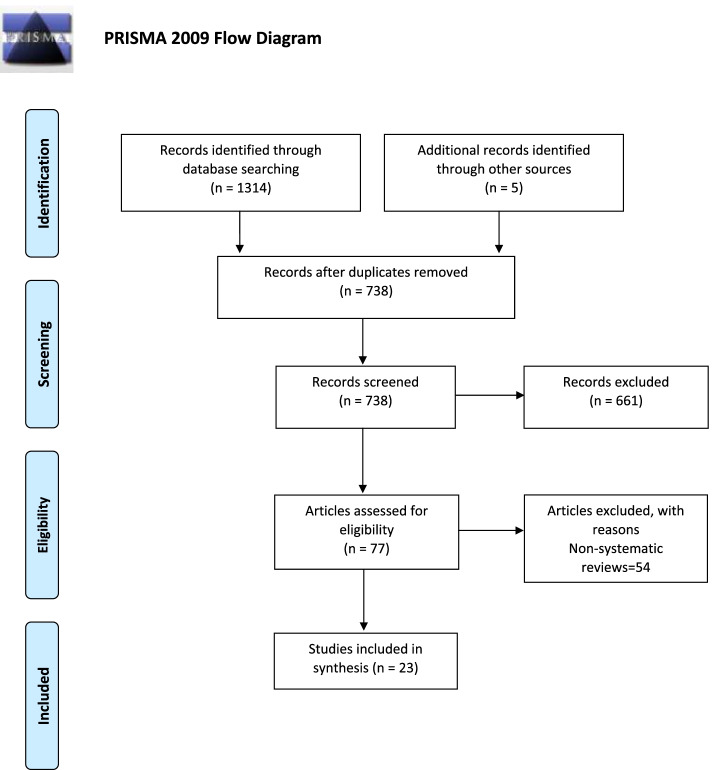
PRISMA flow diagram showing results of search and process of selecting articles for review

Pre-defined inclusion criteria were used to select articles relevant to study objectives identified through the search strategy. Due to the broad nature of scoping reviews, we did not limit our research question to a particular type of article. Indeed, case reports, original articles, guidelines, expert opinions, or posters at conferences/congresses were all eligible for inclusion in order to capture all the results in this area. Based on predetermined criteria, we excluded records investigating non-human samples. Two authors (MV and GN) independently selected the articles according to study question and exclusion criteria. Disagreements were resolved by discussion and the involvement of a third assessor (BB). A consensus was reached in all cases. A relevant number of narrative reviews were selected during the search strategy but were subsequently excluded considering these were referring to one or few previously published reports (i.e., [13, 17, 21]) and, therefore, the strength of the recommendation would have been biased. Therefore, we decided to include only systematic reviews or meta-analyses considering the higher reliability of these methodologies. PRISMA guidelines for scoping review were followed in the selection procedures (Fig. 1). Study quality was not systematically measured; indicators of quality were assessed during selection and have been reported in Tables 1 and 2.

**Table 1:** Results from selected original articles

Author, year	Country	Type of publication	Patients characteristics	BUP indication and dose	BUP effect on depression/other symptoms	Safety considerations/side effects	Additional findings/comments	Study limitations
Ahn, 2018 [29]	Asia (Korea)	Case report	1 pt. (86 yo female), with early-stage PD and depression and comorbid mixed dementia (Alzhei- mer’s disease and vascular).	Depression, 150 mg/day.	Not reported.	Pt developed propriospinal myoclonus (PSM) a few days after BUP introduction and Memantine increase that improved after discontinuation of both drugs.	BUP was suspected to cause PSM considering its effect on suppression of the transport of signals across the synapse in both sympathetic and parasympathetic pathways by antagonizing nicotinic-type acetylcholine receptors, inducing disturbance of autonomic ganglia signals.	Co-administration of Memantine might be responsible for PSM.
Benincasa, 2011 [30]	Europe (Spain)	Case report	3 females (mean age 53.7 years) with PD who developed compulsive eating after dopaminergic medications (ropinirole and pramipexole) with comorbid depressive symptoms (HAMD mean score: 14.7).	Compulsive behavior + depressive symptoms 150 mg/day.	Drastic reduction of food intake within 3–4 weeks and weight loss. Mild improvement on depressive symptoms (HAMD mean score 11.4) at 3-month follow-up.	No significant side-effects nor worsening of parkinsonian motor symptoms (as measured on the UPDRS).	Therapeutic efficacy of BUP on compulsive behaviours may depend on inhibition of dopamine reuptake in the ventral striatum and, consequently, stabilization of dopaminergic transmission in the mesolimbic system + inhibition of norepinephrine reuptake in the prefrontal cortex.	Case report.
Chen, 2007 [31]	North America (the USA)	Original research	National sample of 7868 pts. (mean age 74.4 ± 7.8 years) with depression and comorbid PD compared with pts. without PD, investigated for 12 months after the first visit for depression.	Depression, dose not reported.	Not reported.	Not reported.	Prescription of BUP in patients with depression and comorbid PD: 6.8%.	No data on BUP efficacy and tolerability.
Gebhardt, 2008 [32]	Europe (Germany)	Case report	1 pt. (57 yo female) with PD and comorbid panic disorder, agoraphobia, disabling somatic symptoms (HAMA: 42) and depression (HAMD: 32).	Anxiety and depression, 150 mg/day.	At 16-week follow-up: - improved anxiety (HAMA:19) and depressive (HAMD:17) symptoms - improved global clinical condition (CGI from 6 to 5, GAF from 40 to 52).	No BUP related side effects.	Impaired dopaminergic function due to loss of dopaminergic neurons in the substantia nigra, the limbic system, the prefrontal projections and other brain areas, may contribute to the development of both anxiety and depression in PD.	Case report. Pt started CBT concomitant BUP. Pt on different medications (Levodopa, Benserazide, Rotigotine, Pramipexole, Rasagiline, Mirtazapine). Reduction of anxiety symptoms might be a consequence of the antidepressant effect of BUP.
Goetz, 1984 [21]	North America (the USA)	Original research	20 pts. with PD (mean age 62.3 years, females n = 9), of whom 12/20 with comorbid depression. Study design: 14 patients enrolled in a double-blind parallel protocol, 8 receiving BUP in the first phase and 6 receiving placebo +12 patients received BUP in an open label fashion, 6 as crossovers from the double-blind placebo group and 6 treated outside the double-blind protocol.	Primarily as adjunctive therapy for motor symptoms in PD, secondarily measuring the effect on depression. Mean maintenance daily dose from 340 to 400 mg/day.	At 9 weeks, all patients statistically improved on motor symptoms (NUDS or NYUPDS). 5 of the 12 depressed pts. showed reduction of depression (on “global impression scales for both parkinsonism anddepression”).	Side effects: nausea/vomiting in 8/20 pts., excitement 9/20, restlessness 4/20, postural tremor 1/20, dyskinesia hallucinations 3/20, confusion 3/20.	Antiparkinsonian and antidepressant effects were independent.	No comparison with placebo in all patients. Depression diagnostic criteria and rating scale not specified. Not reported if concurrent antiparkinsonian medication was prescribed at fixed dosage is.
Honkanen, 2019 [33]	Europe (Finland)	Case report	1 pt. (52 yo male) with long history of depression who developed parkinsonism (mild bradykinesia in the left hand, mild slowness in foot-tapping rate, reduced stride length).	Depression, 150 mg/day.	Not reported.	Not reported.	Brain imaging ([123I]FP-CIT SPECT) after 4-weeks discontinuation of BUP resulted normal compared with imaging after 1-week BUP discontinuation. BUP may cause misdiagnosis in brain dopamine transporter imaging, therefore longer (>1 week) washout of BUP may be needed.	Case report. Other concurrent medications: Venlafaxine 225 mg/day, Levodopa 450 mg/day started after the first scan, Agomelatine 25 mg/day started to replace BUP.
Kate, 2013 [34]	Asia (India)	Case report	1 pt. (75 yo male) with PD and comorbid MDD + blood hypertension.	Depression, 300 mg/day.	Not reported.	Hyponatremia (119.5 mEq/L) occurred 18 days after BUP introduction and normalized by 7 days after BUP stop.	-	Case report.
Kaur, 2012 [35]	North America (the USA)	Case report	1 pt. (58 yo female) with PD and comorbid MDD + obesity, hypertension, and hypothyroidism.	Depression, 300 mg/day.	After BUP increase (from 150 to 300 mg/day) and Rasagiline (1 mg/day) introduction, over the period of one year: - significant improvement in neurovegetative symptoms of depression - limited improvement in PD symptoms.	Therapeutic combination was well tolerated without side effects.	Rasagiline in combination with two antidepressants seem a successful approach.	No standardized scale used to measure improvements in depressive or PD symptoms. Additional concurrent medications (venlafaxine 225 mg/day and implantation with deep brain stimulation).
Kim, 2009 [36]	Asia (Korea)	Original research	15 pts. (mean age 57.2 years, 8 females) with PD and comorbid MDD (DSM-IV criteria), treated with BUP for 12 weeks.	Depression, 300 mg/day.	The mean HAMD score was significantly reduced after the 12-week trial (11.12 ± 6.51) compared with baseline (23.11 ± 5.05). No significant change in UPDRS score.	Most common side effects: dizziness, nausea. No pts. reported significant adverse effects including hallucinations and any other confusional symptoms.	-	Open-label study. Small sample size. No full text published (poster conference).
Kim, 2012 [37]	Asia (Korea)	Original research	9 pts. with PD and freezing of gait (FOG), treated with BUP for 12 weeks.	FOG, 300 mg/day.	Mean GABS total score was reduced after the 12-week trial (30.4 ± 3.5) compared with baseline (34.5 ± 5.0), but not at a significant level.	Not reported.	-	Open-label study. Small sample size. No full text published (poster conference).
Kummer, 2006 [38]	South America (Brazil)	Case report	1 pt. (67 yo male) with PD who developed compulsive use of Levodopa and comorbid mood fluctuations (exacerbated during off-episodes) and increased writing activity suggestive of stereotype behaviours + back pain (exacerbated during off-episodes).	Depression, 150 mg/day.	BUP was started after an unsuccessful 12-week trial with sertraline (100 mg): after 8-weeks no changes in depressive symptoms or craving for Levodopa.	Not reported.	-	Case report. Depressive symptoms were strongly correlated to on-off episodes, therefore strictly associated with PD instead of a comorbid depressive symptoms/disorder.
Leentjens, 2000 [17]	Europe (the Netherlands)	Case report	1 pt. (70 yo female) with PD (UPDRS at baseline: 16) and treatment-resistant depression (HAMD: 25).	Depression, started at 150 mg, then 300 mg/day.	Quick and complete remission of depressive symptoms: HAMD at 1 week: 9, at 4 weeks:7, at 7 months: 6. No changes in motor symptoms (UPDRS at follow-up: 7 months, 16), but subjective improvement in hypokinesia.	Self-limitating nausea reported as the only side effect.	-	Case report. Article in Dutch.
Ritter, 1997 [39]	North America (the USA)	Original research	28 pts. (all males, mean age 68 years) with PD treated with Selegiline in association with different antidepressants (including 3 pts. on BUP), aimed to find the safety of different combinations.	BUP indication not specified Dosage range 150–300 mg/day, mean dose 217 ± 76.4 mg/day.	Not reported.	40 Selegiline-antidepressant drug combinations were found (BUP:3, TCAs:25, SSRI:7, trazodone:5). Only one case of serotonin syndrome was reported (with fluoxetine). No patients on BUP discontinued the drug secondary to adverse effects.	BUP is an appropriate first choice with Selegiline therapy.	Retrospective study.
Stein, 1997 [18]	North America (the USA)	Case report	1 pt. (65 yo female) with late-stage PD, severe recurrent depression, psychosis, and chronic pain (diffuse and burning pain at the lower limbs).	Depression, 300 mg/day.	Switching from Paroxetine to BUP and to sustained-release Carbidopa/Levodopa reduced fluctuations in mood and mobility and improved overall functioning but had no effect on pain that was reduced after tramadol introduction.	Not reported.	-	Complex case-report considering policomorbidity. No standardized measure of BUP effect on depression.
Trivedi, 2002 [40]	Asia (India)	Original research	Double-blind randomized controlled study, 23 pts. treated with BUP and 23 with Sertraline.	Depression, 300 mg/day.	After 6 weeks, pts. on BUP showed a significant improvement in depressive (HAMD), global (CGI) and motor symptoms of PD, compared with patients on Sertraline.	Side effect profile with both the drugs was approximately equal.	-	No full text published (poster conference). The effect of important information (e.g., comorbidities, concomitant medications) cannot be ruled out.
Vasile, 2013 [41]	Europe (Romania)	Original research	20 pts. (mean age 64.9 yrs., 8 females) with PD and comorbid MDD (DSM IV-TR), treated with BUP for 6 months.	Depression, 150–300 mg/day.	Compared to baseline, pts. showed a significant improvement in: - depressive symptoms severity (MADRS: −17.6) - global functioning (GAF: +22.1, and CGI-I: −3.5) - severity of PD symptoms (‘non-motor experiences of daily living’ dimension of UPDRS: −13.2) - quality of life (SF-36: total score) and on ‘mental health’ (+12.3), ‘general health’ (+8.2), ‘vitality’ (+7.7), and ‘social functioning’ (+6.2) subscales.	Good tolerability: mild (n = 4) and moderate (n = 3) side effects. The most frequently reported side effects were anxiety, insomnia, and sweating.	-	Open-label study. Small sample size. No full text published (poster conference).
Vegda, 2020 [42]	Asia (India)	Case report	1 pt. (78 yo female) with PD and comorbid depression + comorbid hypertension.	Depression, 150 mg/day.	Not reported.	Pt developed dyskinesias and dystonia (bilateral upper and lower extremity dystonia, buccolingual crisis, left laterocollis, pseudo-macroglossia and severe laryngeal dystonia) 2 days after BUP start that reduced and disappeared after BUP stop.	Dystonia developed after BUP can be considered similar to dopamine-induced peak dose dystonia by its mechanism of increasing the availability of dopamine.	Severe stage of PD (UPDRS:148) with significant motor and non-motor on-off symptoms.
Załuska, 2011 [19]	Europe (Poland)	Case report	1 pt. (78 yo female) with clinical symptoms of PD and depression + possible cognitive impairment (MMSE score 25 and 22 in two subsequent evaluations) + brain imaging suggestive for features of angiogenic multifocal brain lesions and slight cortical atrophy + several comorbid conditions.	Depression, 150 mg/day.	Improvement of depressive symptoms (HAMD from 18 to 7) after 10 days after BUP introduction, in remission after 1-year follow up. Levodopa contributed to the improvement of motor functions (UPDRS motor from 24 to 15 after 3-week treatment).	No psychotic symptoms associated with BUP treatment.	-	Complex case-report considering policomorbidity and brain lesions.

*Legend***:** *BUP* Bupropion, *CBT* cognitive-behavioral therapy, *CGI* Clinical Global Impression-Improvement, *DSM-IV* Diagnostic and Statistical Manual of Mental Disorders, IV ed., *DSM-IV-TR* DSM-IV, Text Revision, *GABS* Gait and Balance Scale, *GAF* Global Assessment of Functioning, *HAMA* Hamilton Rating Scale for Anxiety, *HAMD* Hamilton Rating Scale for Depression, *MADRS* Montgomery-Asberg Depression Rating Scale, *MDD* major depressive disorder, *MMSE* Mini-mental State Examination, *NUDS* North-western University Disability Scale, *NYUPDS* New York University Parkinson Disease Scale, *PD* Parkinson’s disease, *SF-36* Short Form (36) Health Survey, *SPECT* single-photon emission computed tomography, *SSRIs* selective serotonin reuptake inhibitors, *TCAs* tricyclic antidepressants, *UPDRS* Unified Parkinson’s disease rating scale

**Table 2:** Results from selected non-original articles

Author, year	Country	Type of publication	Aim of the study	BUP indication + dose	Main findings related to BUP	Safety considerations/side effects	Limitations
Agüera-Ortiz, 2021 [43]	Europe (Spain)	Consensus of experts	To assess consensus of exerts (in Psychiatry, Neurology, and Geriatrics) about diagnosis and treatment of depression in PD.	Depression, dose not reported.	Experts agreed BUP is an efficacious pharmacological option for depression in PD.	Experts agreed BUP is well-tolerated, especially regarding the absence or a minimal increase in motor symptoms.	Consensus of experts using Delphi methodology and limited number of experts (n = 37).
Mills, 2018 [44]	North America (the USA)	Systematic review and meta-analysis	To review and analyze results of RCTs about efficacy and tolerability of different antidepressants in PD (20 studies, 5 included in meta-analyses).	Depression, dose not reported.	One study on BUP is listed among treatment options for depression in PD, but data on BUP were not further discussed or included in meta-analyses.	Not reported.	Limited data on BUP.
Paumier, 2010 [45]	North America (the USA)	Meta-analysis	To investigate the effect of different antidepressants (including BUP) on PD progression (data from 6 RCTs, including 2064 subjects with PD prescribed antidepressants or placebo)	Not reported.	Antidepressant- treated subjects showed a lower probability of requiring dopaminergic therapy than those not taking antidepressants (HR = 0.6, p < 0.001); effect not specific to a particular class of antidepressant. Mean change in UPDRS scores was significantly lower in subjects treated with atypical antidepressants (including BUP) than those not taking antidepressants (p < 0.05).	Not reported.	Only abstract available (poster conference).
Pena, 2018 [46]	Europe (Spain)	Clinical guidelines	To establish a series of clinical recommendations on the use of antidepressants in patients with PD, based on a systematic review of the literature.	Depression,150–300 mg/day.	Clinicians should consider BUP to treat apathy in pts. with PD (class IV: other studies, including consensus or expert opinion; recommendation U: inadequate or conflicting data).	Not reported.	Only one database (Medline) was searched during the review process.
Weintraub, 2005 [47]	North America (the USA)	Review and meta-analysis	To review and analyze results about antidepressant effect in pts. with PD (27 studies, 11 included in meta-analysis, data from 772 pts. with PD and comorbid depressive symptoms/disorder).	Depression, dose not reported.	One study on BUP is listed among treatment options for depression in PD, but data on BUP was not further discussed or included in meta-analyses.	Not reported.	Limited data on BUP.

*Legend*: *BUP* Bupropion, *HR* hazard ratio, *PD* Parkinson’s disease, *RCTs* randomized controlled trials, *UPDRS* Unified Parkinson’s disease rating scale

### Data charting and collating, summarizing, and reporting results

The research team investigators (MV, GN, BB) collectively developed the data-charting form to determine which variables to extract. This form was revised during meetings throughout the stages of the review, and uncertainty was resolved with periodic team meetings and the involvement of a fourth investigator (BD). The following variables were extracted from each study: author, year and country of publication, characteristics of patients investigated (i.e., number, age, gender, and clinical variables related to PD or comorbid illnesses), Bupropion primarily indication and dosage, results on effectiveness and safety.

## Results

### Search

Our literature search produced 1319 records reduced to 738 after duplicates were removed. Review of titles and abstracts led to the inclusion of 77 articles for assessment; 54 narrative reviews were excluded (Fig. 1). Ultimately, 23 articles were included.

### Description of articles

Selected articles were published between 1984 and 2021 and this result reflects the commercialization (firstly approved as an antidepressant in the United States in 1985) and subsequent use of Bupropion in clinical practice. An increasing trend towards growing publications in this field emerged since then (1984–1995: n = 1; 1996–2007: n = 7; 2008–2021: n = 15). Reports came primarily from North America (n = 8, 34.8%) and Europe (n = 8, 34.8%).

Most selected articles included case reports (n = 11, 47.8%) [17–19, 29, 30, 32–35, 38, 42] for a total of 12 patients with PD and one with parkinsonism. Seven original articles were selected [21, 31, 36, 37, 39–41], most of which were published only in the form of poster presentations [36, 37, 40, 41]. These included interventional studies of which one randomized controlled trial (RCT) (only in the form of poster presentation) [40], one with a mixed design (some patients were compared with a placebo-controlled group while others were treated with an open label fashion) [21], and three with an open-label design (all in the form of poster presentations) [36, 37, 41]. Two cross-sectional studies included a 12-month prospective investigation [31] and one retrospective analysis of clinical charts [39]. Most articles were relatively small in sample (n < 50 patients), except for one study based on a national sample [31], with overall 7868 patients with PD being investigated.

With respect to review articles, two were systematic literature reviews and included a meta-analysis of the results [44, 47]. Another meta-analysis [45] included six RCTs but did not conduct a systematic revision of the literature (only in the form of poster presentation).

Lastly, one article reporting a consensus of experts [43] and one providing clinical guidelines [46] were selected.

Tables 1 and 2 summarize the results that emerged from original and non-original articles, respectively.

### Original articles - randomized controlled trials

Only one double-blind RCT has been conducted thus far [40] which included 46 patients with PD and comorbid depression (according to the Diagnostic and Statistical Manual of Mental Disorders, IV edition criteria) who were divided into two arms: 23 patients treated with Bupropion 300 mg/day and 23 with Sertraline 100 mg/day as the control condition. The main outcomes were to assess the improvement, after a 6-week trial, of depressive symptoms (as assessed on the Hamilton Depression Rating Scale (HAM-D), as well as the efficacy of treatments as measured with the Clinical Global Impression Scale (CGI)) and in relation to motor symptoms (clinically measured). Patients on Bupropion showed a significant improvement in all outcome measures compared with Sertraline, and the side effect profile (clinically assessed) was approximately equal. Despite this being one of the few RCTs conducted in the field, only the abstract was published as a conference poster and, therefore, the role of potential influencing variables (e.g., comorbidities, concurrent medications) and the strictness of the methodology could not be assessed.

The investigation from Goetz and colleagues [21] adopted a mixed design. Indeed, 14 patients were enrolled in a double-blind parallel protocol, 8 of whom received Bupropion in the first phase and 6 received placebo. Moreover, 12 patients received Bupropion in an open-label fashion, 6 as crossovers from the double-blind placebo group and 6 were treated outside the double-blind protocol. The primary aim of this investigation was to assess the efficacy of Bupropion on motor symptoms in patients with PD (n = 20). At the same time, its effects on depressive symptoms were assessed in a specific subgroup of patients (n = 12/20) who manifested depressive symptoms at the baseline. After 9 weeks, all patients reported a significant improvement in motor symptoms (as measured on the North-western University Disability Scale or New York University Parkinson Disease Scale) and 41.7% of the whole sample reported an improvement of depressive symptoms (measured on a generic “global impression scales for both parkinsonism and depression”). Of note, antiparkinsonian and antidepressant effects of Bupropion were unrelated. Reported findings seem to support the efficacy of Bupropion in subjects with PD with or without comorbid depression, while the limited sample size and the lack of a control group limit the confidence in the results. Additionally, the endpoint was set at 9 weeks, which seems quite short for a clinical trial. Side effects were frequent (nausea and vomiting, excitement, restlessness, and postural tremor were dose-limiting in five patients; hallucinations or confusional states occurred as new phenomena in one patient and recurred or resulted to be exacerbated in two subjects; dyskinesia was exacerbated in one patient).

### Original articles - open-label design studies

Two open-label studies specifically investigated the antidepressant effect of Bupropion in patients with PD [36, 41]. Despite the limited sample size (≤20 patients), a significant improvement of depressive symptoms (as measured on the HAM-D [36] or the Montgomery-Asberg Depression Rating Scale [41]) emerged in both studies after a 6-month trial with Bupropion (150–300 mg/day). With respect to other PD symptoms, the study from Vasile and colleagues showed an improvement on the “non-motor experiences of daily living” dimension on the Unified Parkinson’s disease rating scale [41] while the other investigation showed no changes [36]. Additionally, the investigation from Vasile and colleagues [41] showed an improvement in measures of global functioning and quality of life (on the 36-item Short Form survey). With respect to safety, both studies showed a safe profile of Bupropion, with mild and self-limiting side effects being reported (anxiety, insomnia, sweating [41], dizziness, or nausea [36]).

Another 12-week open-label study addressed the use of Bupropion (300 mg/day) on freezing of gait in 9 patients with PD [37]. At the endpoint, freezing of gait (as measured on the Gait and Balance Scale) was reduced, but not at a significant level, and, therefore, authors concluded this medication was not efficacious in PD patients with this motor alteration.

### Original articles – observational studies

Considering safety concerns of monoamine oxidase type B (MAO-B) inhibitors in PD, Ritter and colleagues [39] retrospectively reviewed the clinical charts of 28 patients with PD, who were prescribed Selegiline in association with antidepressants (including 3 patients taking Bupropion), with the aim to investigate the safety of different combinations. Among the investigated combinations (n = 40), only one interaction emerged (serotonin syndrome with Fluoxetine). With respect to Bupropion, authors considered the medication an appropriate first choice in subjects prescribed Selegiline, whereas tricyclic antidepressants and Trazodone may be reserved as second-line treatments [39].

A cross-sectional prospective study collected data from a national database of veterans who attended clinical visits for depression, followed up for the following 12 months to compare different antidepressants in patients with versus without PD [31]. Results showed interesting data on antidepressant approaches in the two subgroups (which had the same chance to start an antidepressant prescription: SSRIs were the most prescribed and the PD group had slightly higher rates to use newer non-SSRI drugs). Bupropion’s prescription accounted for 6.8% of patients with PD, a percentage similar to Venlafaxine (6.8%) but lower compared to other “classic” serotonergic antidepressants (e.g., Sertraline 28.9%) [31].

### Case reports

Eleven case reports describing the use of Bupropion in patients with PD were selected. Bupropion daily dosage varied in a range included in the therapeutic dose (150–300 mg). In all case reports, the compound was used to treat depressive symptoms or Major Depression in patients with PD [17–19, 29, 30, 32–35, 38, 42]. The effects of Bupropion on depressive symptoms were measured, when reported, at different endpoints, from 8 weeks [38] to 1 year [19, 35].

The majority of these reports that measured changes in depressive symptoms (n = 6, 86%) observed a variable degree of improvement of depressive symptoms [17–19, 30, 32, 35], based on a standardized assessment measure (i.e., the HAM-D) [17, 19, 30, 32] or on clinical evaluation [18, 35]. Conversely, the case of a patient with mood fluctuations (i.e., depressive symptoms worsened when Levodopa medication wore off and opposite “euphoric” symptoms manifested during the peak dose) with no improvement after antidepressant treatment, including an 8-week trial with Bupropion, was reported [38]. The remaining case reports (n = 4) did not measure changes in depressive symptoms, as they were primarily focusing on Bupropion’s side effects or on other topics [29, 33, 34, 42].

Some of these reports described Bupropion’s effect on other neuropsychiatric manifestations. Indeed, a case report showed its effectiveness in reducing food intake within 3–4 weeks and promoting weight loss in three patients with PD that developed compulsive eating as a side effect of dopaminergic medications [30]. Another study showed an improvement of anxiety symptoms after a treatment trial with Bupropion [32]. In another study of a woman with late-stage PD, severe recurrent depression, psychosis, and chronic pain (diffuse and burning at the lower limbs) were described [18]. After several unsuccessful treatment strategies to reduce chronic pain and unstable motor and depressive symptoms, switching from Paroxetine to Bupropion – combined with the introduction of sustained-release Carbidopa/Levodopa - reduced fluctuations in mood and mobility and improved overall functioning.

With respect to the safety profile, the majority of cases that reported this measure (n = 7, 70%) confirmed Bupropion overall safety [32, 35] showing no negative motor side-effects [17, 30] or limited improvement in PD symptoms [18, 35]. No psychotic symptoms were registered in a female patient with a positive history of hallucinosis [19], despite the reported risk of this medication to cause this symptom.

On the other hand, some reports (n = 3, 30%) described potential side effects that occurred during the administration of Bupropion. A 78-old patient with a severe stage of PD developed dyskinesias and dystonia two days after Bupropion initiation that reduced and disappeared after its discontinuation [42]. Similarly, Ahn and colleagues described the occurrence of propriospinal myoclonus a few days after Bupropion introduction and Memantine increase, which improved when the two drugs were discontinued [29]. Another case report described a patient with PD and comorbid Major Depressive Disorder who developed hyponatremia (119.5 mEq/L) 18 days after Bupropion initiation; the alteration normalized by 7 days after drug discontinuation [34].

Lastly, one case report described Bupropion’s interference with neuroimaging test in a patient with a long history of depression who developed parkinsonism (mild bradykinesia in the left hand, mild slowness in his foot-tapping rate, and reduced stride length) [33]. In particular, 1 week after Bupropion discontinuation (according to the usual time indicated for washout in imaging centers) the patient performed a brain imaging ([123I]FP-CIT SPECT) which resulted in abnormal (reduced binding bilaterally but particularly in the left putamen) and suggestive for dopamine transporter binding defect. Indeed, a Levodopa treatment trial was started, without clinical response. Eleven months later (this time, four weeks after Bupropion discontinuation) a new SPECT was performed and resulted normal, suggesting that medication might have been the reason for misdiagnosis in the brain dopamine transporter imaging [33].

### Systematic reviews, meta-analyses, and other reports

Despite the vast number of literature reviews focusing on treatment approaches in patients with PD who manifest neuropsychiatric symptoms, only two systematic reviews mentioned Bupropion [44, 47]. Both reports focused on antidepressants use in patients with PD and the review from Mills and colleagues included only RCTs [44]. In these reviews, two articles were included, the one from Goetz, 1984 (in Weintraub, 2005) and the one from Trivedi, 2002 (in Mills, 2018), which have been already discussed the previous paragraph. In both cases, a meta-analysis was performed, but the specific studies on Bupropion were not included in further analyses, so additional data on this medication were not available.

Similarly, Paumier and colleagues [45] conducted a meta-analysis on six RCTs investigating the effect of antidepressants (including Bupropion) on PD progression. This is the only investigation that showed how antidepressant-treated subjects had a lower probability of requiring dopaminergic therapy compared with those individuals not taking antidepressants (HR = 0.6, *p* < 0.001). Of note, this effect was not specific to a particular class of antidepressants. Additionally, mean change in the UPDRS (Unified Parkinson’s disease rating scale) scores was significantly lower (i.e., a lesser degree of motor impairment and disability) in subjects treated with “atypical” antidepressants than those not taking antidepressants (*p* < 0.05). Bupropion was included in this class of “atypical” antidepressants, even though a direct effect of Bupropion cannot be drawn considering how these drugs were grouped (with Mirtazapine and Trazodone).

The “Neurological Association of Madrid”, thanks to a review of the literature and the results of a movement disorder study group survey, issued a series of recommendations on the use of antidepressants in patients with PD [46]. These clinical guidelines state that SSRIs are usually the drugs of first choice, as they are perceived as being well tolerated, having few drug interactions, and being suitable for patients with comorbidities. In relation to Bupropion, the Authors concluded that clinicians should consider it to treat apathy in patients with PD with the following degree of evidence: class IV (data based on other studies, including consensus or expert opinion) and recommendation U (inadequate or conflicting data).

Lastly, using a Delphi methodology, an original article collected the opinion of 37 experts in psychiatry, neurology, and geriatrics on different topics related to depression in PD [43]. Forty-nine items revisiting some fundamental clinical aspects of depression in PD were included, including specific ones on Bupropion (i.e., “the dopamine and norepinephrine reuptake inhibitor Bupropion is effective in PD patients?”; and “Given its PD-specific efficacy and tolerability, Bupropion is a good treatment option for depression in PD patients?”). Seeing experts’ agreement, this medication was considered an efficacious and well-tolerated pharmacological option for depressive symptoms in PD [43].

## Discussion

### Effectiveness on depressive symptoms

Considering the results of the present scoping review, the first question that can be answered relates to Bupropion antidepressant effectiveness in patients with PD. Overall, most of the retrieved reports seem to support its use. Indeed, the RCT [40] and three reviewed open-label studies [21, 36, 41] demonstrated a variable degree of the antidepressant effectiveness of Bupropion in patients with PD. Similarly, the majority of case reports who measured depressive outcomes [17–19, 30, 32, 35] showed improvement of depressive symptoms, while only one [38] reported no changes. Despite diverse methodology (e.g., different measures of depressive symptoms, different Bupropion dosage, and duration of therapy) and publication biases (i.e., negative findings remain often unpublished [48]), we can conclude that Bupropion is potentially a valuable treatment option to target depressive symptoms in patients with PD. This recommendation is supported by the treatment guidelines from the Neurological Association of Madrid [46], specifically indicating Bupropion for the treatment of apathy associated with PD, though with a low level of evidence and grade of recommendation (class IV, recommendation U). Apathy is a common symptom in PD patients, with a frequency reported between 16.5% and 42% of cases of PD [49]. According to these guidelines, Bupropion is the only antidepressant recommended for apathy: thus, it might be particularly useful in PD patients who manifest an intense lack of feelings, emotions, or interests.

Similarly, Aguera-Ortiz and colleagues [43] showed that clinicians considered Bupropion as an efficacious and safe pharmacological option in the treatment of depression in PD patients. Available treatment guidelines about depression in PD [50] are inconclusive and underline the lack of robust literature data, as emerged in the systematic reviews included in the present paper [44, 47]. Therefore, clinicians presumably base their therapeutic decisions on clinical experience, which seems to favor Bupropion use in PD patients with depressive symptoms.

### Effectiveness on motor symptoms

Considering Bupropion’s pro-dopaminergic effect, its potential parallel role in ameliorating motor symptoms in PD is the second issue that can be discussed in light of the results of the present review. Among selected articles that measured motor outcomes, the two RCTs [21, 40], one original article [41] and two case reports [18, 35] showed an improvement in motor symptoms following treatment with Bupropion. Additionally, using a meta-analytic approach, Paumier reported that, in subjects with early PD, antidepressant therapy (including but not specific on Bupropion) reduced the probability of requiring dopaminergic therapy compared with subjects not taking antidepressants [45]. However, these investigations adopted questionnaires that measured generic changes in motor symptoms, so the effect of Bupropion on a specific PD-related motor symptom cannot be surveyed. Only the investigation from Kim and colleagues [37] specifically showed a reduction of gait freezing in PD patients treated with Bupropion, although this difference was not significant when compared with baseline. In this respect, although the exact mechanism of action of Bupropion is not fully understood, this improvement in motor symptoms is likely associated with increased dopaminergic levels in the dopaminergic pathways involved in PD [51].

On the other hand, among investigated studies, two case reports reported dyskinesias and dystonia [42] and propriospinal myoclonus [29] occurring after Bupropion start: these adverse events improved and disappeared after its discontinuation. Indeed, they might be considered similar to dopamine-induced peak dose dystonia given Bupropion’s mechanism of increasing the availability of dopamine [52] or, in the circumstance of myoclonus, possibly produced by the concomitant use of Memantine [53].

Overall, the present scoping review seems to support a certain degree of efficacy of Bupropion also on motor symptoms, even though additional investigations with larger samples and with a strict methodology are needed to confirm this conclusion. In the attempt to analyze motor side effects in patients on Bupropion, a recent systematic review (non specific on PD patients, with Bupropion prescribed for different reasons) collected 710 cases describing Bupropion-associated movement disorders. The three most commonly reported were tremors, slurred speech, and falls, but also dystonia, dyskinesia, parkinsonism and myoclonus were variably reported. However, the Authors concluded Bupropion was uncommonly related to abnormal movements and that the majority of literature cases did not clearly report the clinical neurological examination and lacked electrodiagnostic tests: therefore, the reported alterations might be not primary a consequence of Bupropion [24].

### Effectiveness of Bupropion on other neuropsychiatric manifestations

In the general population, Bupropion is approved for depression, seasonal affective disorder, and smoking cessation, while off-label uses often include antidepressant-induced sexual dysfunction, attention-deficit/hyperactivity disorder, depression associated with bipolar disorder, and obesity [54]. In the context of PD, the articles examined in the present review supported Bupropion use to reduce compulsive eating as a consequence of dopaminergic medications [30] and to improve panic symptoms [32]. Although these results represent solitary findings, they might reflect the pharmacodynamics of Bupropion. Indeed, the inhibition of dopamine reuptake in the ventral striatum might lead to a stabilization of dopaminergic transmission in the mesolimbic system (as observed in nicotine addiction where Bupropion showed to be effective) [55]. Moreover, Bupropion-mediated inhibition of norepinephrine reuptake in the prefrontal cortex may contribute to further stabilizing cortical-subcortical prefrontal limbic circuitries involved in addictive and compulsive behaviors. The same neurotransmitter might be responsible for the anxiolytic effect, even though an improvement of anxiety symptoms as secondary to improvement of depression might be another potential explanation.

### Safety profile

The third important issue addressed in the present review is about Bupropion safety in patients with PD. This medication is contraindicated in patients with epilepsy and eating disorders, and the most common side effects occurring in more than 10% of the patients are headache, dry mouth, nausea, weight loss, insomnia, agitation, dizziness. However, Bupropion lacks typical antidepressant-associated side effects such as sexual dysfunction, weight gain, and sedation [56].

The motor side effects described in the reviewed articles have been already discussed in a previous paragraph. Considering non-motor side effects, Bupropion has been considered a safe medication by the majority of the articles [17, 19, 30, 32, 35, 36, 39–41]. Additionally, one investigation showed an improvement in measures of global functioning and quality of life as secondary to Bupropion treatment [41]. Moreover, Bupropion was considered to be safe in combination with Selegiline therapy [39]. Another reviewed case report did not show an increased risk of psychotic symptoms in a female patient with a positive history of hallucinosis [19], whereas hallucinosis was reported in 3 patients (15%) in another selected paper [21]. Some previous reports alerted on the risk of psychosis associated with Bupropion, with incomplete, and in some cases mixed, results as observed by a systematic review of the literature [57]. The side effects reported in selected articles were anxiety, insomnia and sweating [41], dizziness [36, 37], nausea [17, 21, 36], vomiting, excitement and restlessness, postural tremor, dyskinesia, hallucinations, and confusion [21]. Another report described the occurrence of hyponatremia in a PD patient on Bupropion, which was found to be resolved after Bupropion withdrawal [34]. Hyponatremia is reported to be associated with the use of various antidepressants, especially SSRIs, and in 0.41% of subjects prescribed Bupropion in a phase IV clinical study analysis [58].

Overall, Bupropion use is likely associated with a favorable safety profile in subjects with PD, considering the absence or the nonsignificant nature of side effects that emerged in reviewed articles. Apart from specific contraindications, Bupropion might be cautiously used in PD patients with a positive history of psychotic symptoms. Once more, the nature of the reviewed reports and the limited number of included patients must be considered. The work from Agüera-Ortiz supports this recommendation, with a consensus of experts about the good tolerability of Bupropion in patients with PD [43].

## Limitations

Potential study limitations must be kept in mind. First, our search may not have been exhaustive, despite using multiple databases and grey literature sources. Using a systematic review methodology was not possible (considering the limited investigations on the topic); however, the present scoping review has been conducted after a systematic revision of the literature and according to a strict methodology (i.e., PRISMA Guidelines). Second, the authors decided to not include narrative reviews, commentaries, and perspectives which represent a considerable number of sources, but, as already stated, are based on limited reports. Third, a quality assessment of the selected studies was not conducted with a standardized tool, but the authors critically evaluated and indicated in Tables 1 and 2 the most relevant methodological limitations. Lastly, the 22% (N = 5) of records selected were missing the full-text article being posters at conferences/congresses. This issue potentially reflects a limitation of the current knowledge on this topic as it seems that some investigations were conducted and published only as poster presentations/conference abstracts but not in the extended form, likely due to bias that did not allow publishing the trials or other reasons that we are not aware of. Despite the lack of the full-text might limit the confidence in the results, this more inclusive approach is in accordance with the nature of a scoping review, that includes researches and publications that would be characterized as having lower levels of evidence in hierarchies but, on the other hand, includes the kind of evidence that inform clinicians in the decision-making [59, 60] and design of future RCTs needed to produce accurate clinical guidelines.

## Conclusion

**Fig. 2:**
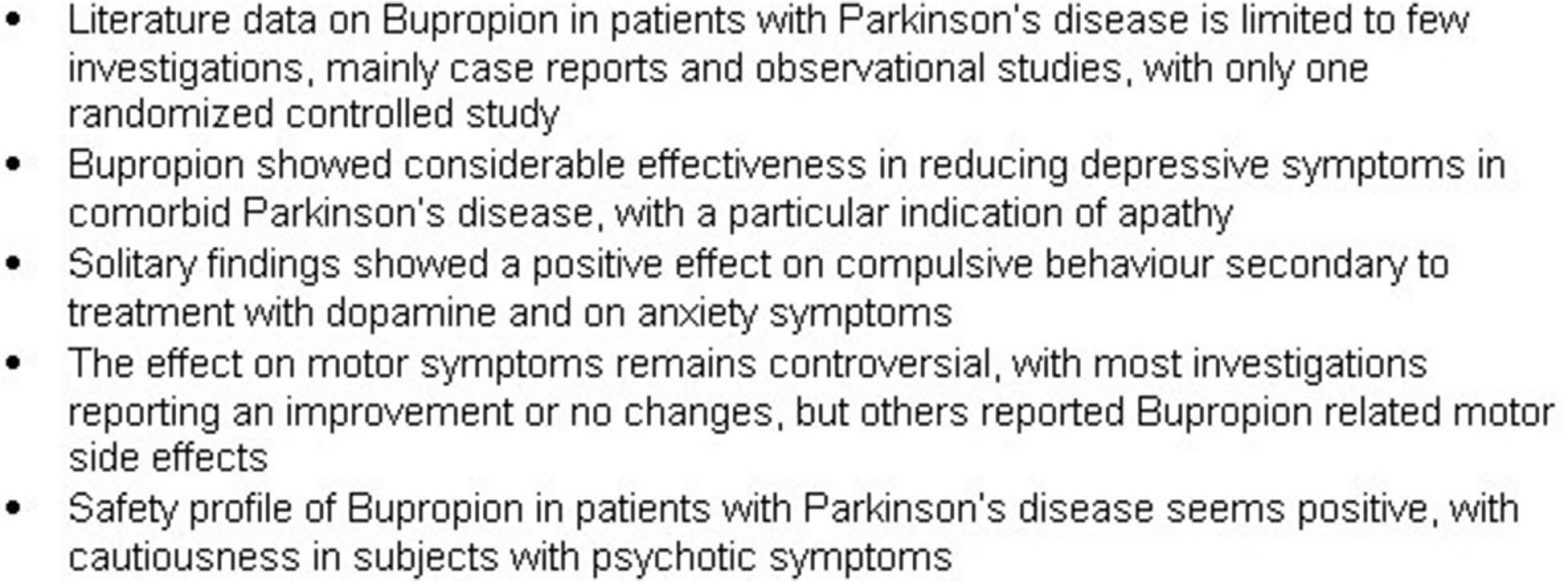
Main findings emerged in the present scoping review

The present scoping review sought to provide a comprehensive and updated overview of Bupropion clinical uses in patients with PD who manifested depression or other neuropsychiatric symptoms. Figure 2 describes the main findings and related recommendations that emerged from the present work.

Considering the current literature limitations and the scarce number of patients with non-motor symptoms treated with Bupropion, it was not possible to stratify them according to specific disease variables, like severity, duration, or pharmacotherapy. However, we tentatively delineated a patient’s profile more suitable for treatment with Bupropion. Patients with PD and depressive symptoms in particular apathy seem to favor the use of this medication, which should preferably not be used in subjects who present a history of psychosis and in ones with a long history of PD or unstable response to treatment with dopamine.

Considering the unique mechanism of action of the medication and the encouraging results emerged in the present scoping review, further investigations in this area, in particular RCTs with larger sample sizes, are encouraged and needed to overcome current literature limitations and to better understand the efficacy and safety profile of the compound in this specific population.

## Data Availability

All data generated or analyzed during this study are included in this article/manuscript.
